# Efficacy of a Short-Term Lifestyle Change Intervention in Healthy Young Men: The FASt Randomized Controlled Trial

**DOI:** 10.3390/ijerph20105812

**Published:** 2023-05-13

**Authors:** Francesco Donato, Elisabetta Ceretti, Gaia Claudia Viviana Viola, Monica Marullo, Danilo Zani, Stefania Ubaldi, Sabina Sieri, Stefano Lorenzetti, Luigi Montano

**Affiliations:** 1 Unit of Hygiene, Epidemiology and Public Health, and Unit of Urology, Department of Medical and Surgical Specialties, Radiological Sciences and Public Health, University of Brescia, 25123 Brescia, Italy; francesco.donato@unibs.it (F.D.); gaia.viola@unibs.it (G.C.V.V.); monica.marullo@unibs.it (M.M.); danilo.zani@unibs.it (D.Z.); 2 European Lifestyle Medicine Organization (ELMO), 1201 Geneva, Switzerland; stefania.ubaldi@amge.ch; 3 Swiss Society of Lifestyle Medicine (SSLM), 1204 Geneva, Switzerland; 4 Epidemiology and Prevention Unit, National Institute for Cancer, 20133 Milan, Italy; sabina.sieri@istitutotumori.mi.it; 5 Department of Food Safety, Nutrition and Veterinary Public Health, Italian National Institute of Health (ISS), 00161 Rome, Italy; stefano.lorenzetti@iss.it; 6 Andrology Unit and Service of Lifestyle Medicine in UroAndrology, Local Health Authority (ASL) Salerno, Coordination Unit of the Network for Environmental and Reproductive Health (EcoFoodFertility Project), Oliveto Citra Hospital, 84124 Salerno, Italy; l.montano@aslsalerno.it; 7 PhD Program in Evolutionary Biology and Ecology, University of Rome Tor Vergata, 00133 Rome, Italy

**Keywords:** adolescence, dietary habits, lifestyle intervention, Mediterranean diet, physical activity

## Abstract

The aim of this study was to investigate the impact of dietary habits and physical activity intervention on lifestyle behavior as a prevention tool supported also by personalized motivational counseling. A two-arm randomized controlled trial was carried out. A sample of 18–22-year-old students was randomly assigned to a four-month intervention based on the Mediterranean diet and moderate physical activity program (N = 66) or to a control group (N = 63). The outcomes were adherence to the Mediterranean diet, physical activity level, and nutrients intake, assessed at enrollment (t0), end of intervention (t4, 4 months after the start), and end of follow-up (t8, 8 months after the start). Adherence to the Mediterranean diet increased from t0 to t4 and t8, more in the intervention (6.83, 9.85, and 9.12, respectively) than in the control group (6.73, 7.00, 7.69, respectively) (*p* < 0.001). Physical activity showed a moderate increase from t0 to t4 and t8 in both groups, without significant differences between them. Significant differences were seen between the two groups in food intake changes, from t0 to t4 and t8. This randomized controlled trial showed that a moderate short-term intervention based on the Mediterranean diet and regular physical activity determined a positive change in the lifestyle of healthy, normal-weight, young men.

## 1. Introduction

The primary prevention of chronic, non-communicable diseases has become a priority of public health policies in both high- and low-income countries [1]. An incorrect diet and physical inactivity are among the main causes of chronic diseases in the world, according to the Global Burden of Diseases Study [2]. Healthy diets from sustainable food systems have been proposed for health promotion and prevention of chronic diseases, in agreement with the UN Sustainable Development Goals and the Paris Agreement [3]. Particularly, the Mediterranean diet, characterized by a high intake of vegetables, legumes, fruits, nuts, grains, fish, and extra virgin olive oil, has been associated with a reduced risk of developing chronic diseases in both experimental and observational studies [4,5]. In addition, moderate and regular physical activity has been associated with positive health effects and is recommended by the World Health Organization (WHO) and many Agencies and Scientific Societies [1,6,7].

Most trials on lifestyle interventions have been performed in adults, especially in those at higher risk of developing metabolic and cardiovascular diseases. However, adolescents are a target population for health promotion interventions, because of the opportunity for acquiring positive lifestyle habits at an early age to be maintained in adulthood [8]. Various trials have been carried out among overweight and obese children and adolescents and in those with the metabolic syndrome, for prevention of the subsequent development of chronic diseases [9,10]. According to a Public Health perspective, however, healthy lifestyle education programmes should be addressed not only to individuals at higher risk for metabolic and cardiovascular diseases, but also to the general population [11]. To our best knowledge, no randomized controlled trial has been carried out on the effects of dietary and physical activity interventions in healthy young men with normal body weight so far.

This study, part of a large randomized trial study on semen quality of healthy young men in Italy (the FASt project) [12], had the specific aim to investigate the impact of dietary habits and physical activity intervention on lifestyle behavior as a prevention tool in healthy normal-weighted young men. In order to maximize adherence to recommended lifestyle changes, personalized motivational counseling has been also performed in the intervention group.

## 2. Materials and Methods

### 2.1. Study Design

This is a 1:1 randomized controlled trial (RCT) (registered on the ClinicalTrials.gov Protocol Registration and Results System (PRS), Receipt Release Date: 15 February 2019, n. J59D1600132001) which aims to evaluate the short-term effects of a lifestyle intervention on the dietary habits and physical activity of healthy young men living in the Brescia province.

### 2.2. Inclusion Criteria, Participants, Ethics

The subjects were enrolled among high school and university students aged 18–22 years. To this end, we held 20 min meetings with students in each class, during a lesson, in the presence of their teacher, to explain the study design and invite the students to participate. Since this trial was part of a study on semen quality and environmental risk factors in healthy adolescents living in polluted areas in Italy [12], we enrolled only males, according to the following inclusion criteria, which aimed to limit the effect of possible risk factors for semen quality, such as tobacco smoking, alcohol drinking, overweight/obesity and others: body mass index (BMI) between 18.5 and 25, waist circumference < 102 cm, no regular use of steroids or anabolic hormones, no or low intake of alcohol (<5 drinks per week), no or low tobacco smoking (<5 cigarettes, cigars, or pipes per week), no or low marijuana smoking (<3 times a month), and no regular use of other drugs or medicine.

After filling in a short self-administered screening questionnaire for excluding subjects not suitable to the research, the potential candidates were invited to undergo a urologic visit, including a clinical examination and spermiogram analysis, by a specialist physician, in a Urology and Fertility Unit. At the same time, participants underwent a fasting blood sample for the analysis of some common parameters (glycemia, cholesterolemia, etc.), and measurement of height, weight, and abdominal circumference by two trained nutritionists, using standardized instruments and methods [13].

Upon their recruitment, all subjects filled in three questionnaires on their dietary habits and physical activity, using standardized questionnaires, under the control of a nutritionist. The questionnaires were administered three times: at the enrolment (t0), 4 months (t4), and 8 months after baseline evaluation (t8).

All the participants were also interviewed on their medical history and provided a blood sample for the analysis of common parameters, as indicators of possible unrecognized disease: fast glycaemia, protein levels, blood cell counts and characterization, etc. Only those with absence of severe disease and normal blood parameters were included in the study.

The study was conducted according to the guidelines of the Declaration of Helsinki. The protocol was approved by the Ethic Committees of Southern Campania (29 November 2017), the coordinating center of the FASt study, and by that of Brescia Province (13 March 2018), and accepted by the Italian National Institute of Health (20 December 2017).

All the participants signed an informed consent form. Participation was on a voluntary basis and participants were free to withdraw from the project and revoke their consent to use their data at any time. All the data analyses were carried out in an aggregate and anonymous way. The data were collected and analyzed in accordance with Legislative Decree No. 196 of 30/6/2003 “Personal Data Protection Code” and the new European Data Protection Regulation 2016/679 (EU).

The study started with the recruitment and first evaluation of the participants between April 2018 and January 2019 (t0) and ended in November 2019.

### 2.3. Assessment of Diet and Physical Activity

The participants’ dietary habits were investigated using two questionnaires. The “PREvención con DIeta MEDiterránea” (PREDIMED) questionnaire consists of 14 questions, which assess the frequency of intake of foods typical of the Mediterranean diet and of those of the “Western” diet, providing an overall 0–14 score [14]. A score of less than or equal to 7 indicates a low adherence, while a score greater than 7 indicates a high adherence to the Mediterranean diet. The PREDIMED questionnaire was self-completed by the recruited subjects in a paper form.

For evaluating the dietary intake of macro- and micro-nutrients for each subject, we used the European Prospective Investigation into Cancer and Nutrition (EPIC) questionnaire, a Food Frequency Questionnaire (FFQ) developed for the EPIC project [15] and validated in its Italian version [16]. The EPIC questionnaire evaluates the frequency of food intake in a one-year period, and the whole daily or weekly intake of nutrients such as fiber, protein, lipids, and carbohydrates. The EPIC questionnaire was completed by each subject with the support of the nutritionists in an online form. The results have been read and processed by the Milan National Cancer Institute Diet research group, which developed a software and a food classification system to analyze and extrapolate information from the questionnaire [17].

Physical activity was evaluated using the International Physical Activity Questionnaire (IPAQ) [18,19], which calculates the Metabolic Equivalent of Tasks (METs), expressed as the ratio of oxygen consumption during a given physical activity to that of the sitting and resting position. Physical activity level was estimated by multiplying the duration of the activity (in minutes) for the days on which it took place during the last week and the METs specific for each type of activity. Based on the overall score obtained, subjects were classified into 3 categories: inactive subjects (<700 METs), sufficiently active subjects (700–2519 METs), active or very active subjects (≥2520 METs).

### 2.4. Intervention

The dietary intervention was a step-by-step educational pathway, intended to encourage participants’ adherence to a Mediterranean diet pattern, provided by trained nutritionists in individual meetings. Accordingly, the latest version of the Mediterranean food pyramid was used to build up a model of intervention, to achieve the following goals:

- Daily vegetables and fruit intake (possibly seasonal and organic)
- Increase pulses intake up to 3/4 times a week
- Increase fish intake up to 3/4 times a week (particularly oily fish)
- Encourage nuts intake, up to 30 g a day
- Encourage consumption of extra virgin olive oil
- Encourage consumption of whole grains and their products
- Limit cheese consumption twice a week (milk, yoghurt and kefir were allowed every day)
- Limit meat intake to 3/4 times a week (2/3 times white meat, once a week red and processed meat)
- Limit alcohol consumption to a maximum of 2 alcohol units a day
- Limit desserts and sweet snacks to once a week

After having collected the subject’s dietary habits using the EPIC questionnaire, the nutritionist proposed an individualized diet to each participant, based on some specific goals. The nutritionists developed a brief booklet, introducing the Mediterranean diet and describing its main food categories, as well as their suggested intakes. The booklet was provided to each subject of the intervention group during the first meeting. A regular and moderate physical activity of at least 150 min every week was also recommended if the participant did not play sports regularly.

Various models have been proposed for counseling for behavior changes to prevent no adherence to recommended lifestyle changes, which is a frequent and difficult obstacle to achieve significant results [20]. In this study, the individual lifestyle intervention was designed on the Motivational Interview (MI) model. Briefly, MI is a collaborative, goal-oriented style of communication, designed to strengthen personal motivation for and commitment to a specific goal by eliciting and exploring the person’s own reason for change [21]. MI is designed to empower people to change by bringing out their own strengths, significance, importance, and their ability to change. It is based on a respectful and curious way of being with people, which facilitates the natural process of change, while respecting autonomy. Due to the young age of the participants, the nutritionists carried out the interviews according to the Naar-King and Suarez’s suggestions in “The motivational interview with the adolescent” [22]. They established in the first meeting a few simple objectives for each subject, according to his personal food preferences, to foster autonomy and collaboration and to evoke motivation to change. In order to encourage participation and limit attrition, the participants of the intervention group received some samples of organic food during the trial, which were in line with our indications for a healthy diet (olive oil, tomato juice, wholemeal pasta and crackers, and so on). Third, the nutritionists contacted each participant by telephone weekly to address any doubt or difficulty in achieving the proposed objectives. Finally, the nutritionists carried out monitoring meetings with each participant, for checking and updating his dietary habits, following the Miller and Rollnick’s management protocol [23], according to the following schedule:

- 1st month: once a week
- 2nd and 3rd months: every two weeks
- 4th month: at the end of the month (final examination)

To keep the participants motivated to achieve their individualized goals, they were asked to record their food intake in a diary during the last 30 days.

The control group received only a leaflet reporting a graphic representation of the Mediterranean diet pyramid and generic recommendations for a correct diet by the Italian Council for Agricultural Research and Economics [24] during the first meeting with the nutritionist.

### 2.5. Sample Size

The sample size of this two-arm parallel randomized controlled trial was established to observe a statistically significant difference of at least one point in the PREDIMED score (standard deviation (S.D.) = 2, effect size = 0.5) between the experimental and control groups at the end of the intervention time (4 months after first visit), while adjusting for the baseline value as a covariate by analysis of covariance (ANCOVA), assuming a minimum correlation coefficient of 0.4 between the baseline and end of the study value in the control group. According to a 1:1 randomization plan, an enrollment of 70 subjects per group, assuming a maximum of 20% loss of subjects for the end of the intervention time, would have provided more than 80% power for statistically significant ANCOVA for testing the between-groups difference at the threshold of 0.05.

### 2.6. Randomization and Blinding

Participants were randomly assigned to either the lifestyle intervention group or the control group in a 1:1 ratio, using a computer-generated randomization list. A statistician, assigning a code to each subject, performed the allocation. Due to the study design, both participants and researchers were aware of the intervention assignment. However, the extraction of data from the questionnaires by the Milan National Cancer Institute Diet research group and the statistical analysis were performed blindly.

### 2.7. Data Management and Analysis

All the data were collected in a database. The analysis of efficacy of the trial was made per protocol and not by intention to treat, because not all the outcome variables were available for dropouts. Descriptive statistics, including means and S.D., range and proportions, whenever appropriate, were reported for each group separately.

Analysis of covariance (ANCOVA) with a baseline value as a covariate was performed for testing the changes in PREDIMED, IPAQ, and EPIC data between the baseline (t0) and end of the intervention (t4) and between the baseline (t0) and end of follow-up times (t8), as it is one of the most effective methods for the analysis of pre–post continuous measures in randomized controlled trials [25].

All the statistical tests were two-sided with a threshold of 0.05 for rejecting the null hypothesis. Data analysis was carried out using the Stata 14.2 software (Stata Corp, College Station, TX, USA).

## 3. Results

**Figure 1:**
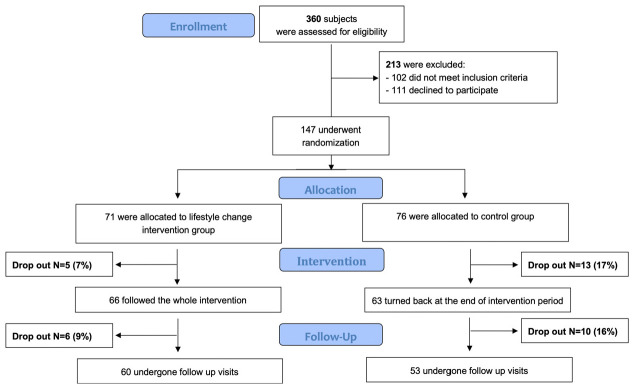
Consort flow diagram of the study.

Of the 360 students assessed for eligibility, 213 were excluded because they did not meet the inclusion criteria (some of them reported a history of chronic disease or had abnormal blood values, indicative of possible disease) or declined to participate (Figure 1). Therefore, 147 students underwent randomization and were assigned to the lifestyle change intervention (INT) (N = 71) group or the control (CTRL) (N = 76) group. A total of 129 subjects (88%) completed the visit and filled in the questionnaire at the 4-month visit (66 INT and 63 CTRL) and 113 (77%) underwent also the final follow-up visit at 8-month (60 INT and 53 CTRL). Most reasons for withdrawal were family issues and other commitments that were unrelated to the study. The 129 subjects with complete data before and after the intervention (t0 and t4) were included in the main statistical analysis.

Baseline characteristics of the participants are reported in Table 1. The mean age of the subjects was 20 years. According to the inclusion criteria, all subjects were in the normal BMI and waist circumference range; among them, 6.2% were occasional smokers and 71.3% light, occasional alcohol drinkers. No statistically significant difference was found between subjects in the INT and CTRL groups for any variable.

Adherence to the Mediterranean diet was low at baseline, with the majority of subjects (57.6% and 65.1% in the INT and CTRL groups, respectively) reporting a low adherence score at the PREDIMED questionnaire stage (≤7). Most subjects were referred to a high level of physical activity in both groups according to the IPAQ score (>700 METs) (87.1% and 80.3% in the INT and CTRL groups, respectively). Again, no statistically significant difference was found between the two groups.

**Figure 2:**
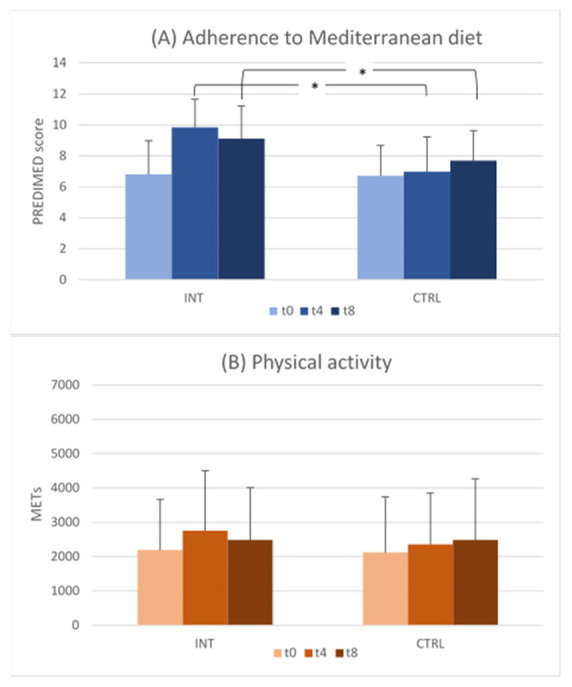
Mean score and S.D. of adherence to (**A**) Mediterranean diet (PREDIMED questionnaire) and (**B**) physical activity (IPAQ questionnaire) in the intervention (INT) and control (CTRL) groups, at baseline (t0), end of intervention (t4), and end of follow-up (t8). For PREDIMED, at t0 and t4, INT: N = 66 and CTRL: N = 63. At t8, INT: N = 60 and CTRL: N = 53. For IPAQ, at t0 and t4, INT: N = 62 and CTRL: N = 61. At t8, INT: N = 53 and CTRL: N = 52. *: *p* < 0.001 for t0–t4 and t0–t8 comparisons between the INT and CTRL groups for the PREDIMED score; *p* > 0.05 for the t0–t4 and t0–t8 comparisons between the INT and CTRL groups for the IPAQ score in METs (ANCOVA).

The PREDIMED mean score increased from t0 to t4 and t8, more in the INT (6.83, 9.85, and 9.12, respectively), than the CTRL group (6.73, 7.00, and 7.69, respectively) (*p* < 0.001 for both t0–t4 and t0–t8 comparisons between the INT and CTRL groups by ANCOVA) (Figure 2A). The IPAQ mean score showed a moderate increase of physical activity from t0 to t4 and t8 in both groups, without significant differences between them (Figure 2B).

The food intakes at t0, t4, and t8 are shown for each group in Table 2. The intake of vegetables, legumes, fruits, nuts and seeds, whole grain, and fish increased, and that of red and processed meat, cheese, sweets, and sweetened drinks decreased, from before to end of the intervention (t0 vs. t4), significantly more in the INT than the CTRL group. Most of the differences in food intake between the two groups were reduced but still statistically significant at t8.

Macro- and micro-nutrient intakes at t0, t4, and t8 for both groups are shown in Table 3. In line with the food consumption changes seen in Table 2, the intake of total protein, particularly animal protein, total saturated fats, and sodium decreased, and that of plant protein, omega-3 and omega-6 fatty acids, fibers, vitamin C, folic acid, beta-carotene, and vitamin E increased, from t0 to t4, significantly more in the INT than the CTRL group. Only a part of these differences between the two groups remained statistically significant at t8.

Two comparisons between the subjects attending and those not attending the 4th- and 8th-month (t4 and t8) evaluations were performed, for each variable and dietary parameter (food groups and nutrients) at baseline. No significant difference was found between participating subjects and the dropouts of the control group at the 4th-month evaluation for food items or nutrients, using the nonparametric Mann–Whitney test, at the reduced threshold of *p* = 0.01 for rejecting the Null hypothesis, due to the multiple comparison problem (data not shown in Tables). No comparison could be done between participants and nonparticipants at the 4th-month evaluation in the intervention group, due to the small number of nonattending subjects (n = 5). Accordingly, the comparison between the attending and nonattending participants for the 8th-month evaluation (t8) did not show substantial differences between them for almost all baseline values of investigated variables, in both the intervention and control groups (data not shown in Tables).

## 4. Discussion

This randomized controlled trial showed that a 4-month lifestyle intervention based on the Mediterranean diet and moderate physical activity determined a positive change in diet quality and physical activity in healthy, normal-weight, young men.

The subjects enrolled in the intervention group, who followed a nutritionist-counseled pathway based on a Mediterranean diet pattern for 4 months, modified their habits, on the average, in a relatively short time (4 months), compared to those in the control group, who received only general recommendations on a correct lifestyle. The latter approach was considered equivalent to a “placebo”, as it has not been found to modify individuals’ habits according to literature.

As regards diet, we observed significant differences in food intake changes, from the beginning to the end of the intervention time, between the two groups. Particularly, the mean intake of vegetables, legumes, fruits, nuts and seeds, whole grains, and fish increased, and that of red and processed meat, cheese, sweets, and sweetened drinks decreased significantly more in the INT than the CTRL group. Accordingly, the mean intake of animal protein, saturated fats, and sodium decreased, and that of plant protein, omega-3 and omega-6 fatty acids, fibers, vitamin C, folic acid, beta-carotene, and vitamin E increased in the INT group, with statistically significant differences as compared to the CTRL group. In agreement with the changes in the macro- and micro-nutrient intake, also the PREDIMED mean score increased significantly more in the INT than the CTRL group.

Most of the significant differences observed between the INT and CTRL groups at t4 were still present at t8, 4 months after the end of the intervention, although a decline in the adherence to dietary suggestions was found as regards the PREDIMED score and food items. In the same way, the mean physical activity level, measured as the IPAQ score, showed a slight decrease from the end of the intervention to the end of follow-up (from 4 to 8 months since the start) (see Figure 2). A decline in the adherence to lifestyle suggestions after the end of an educational intervention is expected. Indeed, the behavioural changes imply a maintenance phase, with possible relapses to pre-intervention status [23]. Therefore, various activities, including support from people with healthy behaviours and from other sources, including family, may be needed for maintaining healthy lifestyle changes over time.

The control group showed a lower change in dietary habits than the intervention group, particularly at the 4-month evaluation. However, the control group increased physical activity, similarly to the intervention one, without substantial differences between the two groups. Overall, these findings suggest that participation in the study by itself, with the visits, blood sampling, and interviews, was a sort of “nudge” for the students in the control group, in spite of receiving no more than a leaflet and generic recommendations to follow present guidelines. Furthermore, since both intervention and control group participants were students in the same classes, a potential contamination between the two groups may have occurred, with some control group subjects following the suggestions for the intervention group. Therefore, the “true” efficacy of the intervention may be higher than that which we found when comparing the before–after changes between the two groups (“dilution effect”).

The age mismatch between our study and other diet-based RCTs makes a comparison difficult. Usually, RCTs on diet are conducted among adults or old people and focused on making people lose weight, have better control of their chronic diseases, such as hypertension, diabetes, or metabolic disease, or prevent chronic disease occurrence. Overall, dietary intervention studies showed that an increased adherence to the Mediterranean diet has benefits in reducing body weight and/or risk of diabetes, metabolic syndrome, cardiovascular disease, and/or in contrasting risk factors for chronic diseases [26,27,28,29]. Nevertheless, a few studies evaluated the effects of dietary interventions in healthy adolescents. A cluster-RCT study on 9–18-year-old students found that a 12 h health promotion intervention significantly increases adherence to healthy diet guidelines, in particular regarding the 5-a-day fruit and vegetable consumption, even after a 4.5-year follow-up period [30]. Another RCT showed the positive impact of a website promoting healthy nutrition for adolescents as regards the vegetable intake [31]. In their clustered-RCT, Chamberland and colleagues [32] found that their web-based nutrition program increased the consumption of vegetables and fruit in healthy high school students, although the effects disappeared 10 weeks after the intervention. However, none of these studies evaluated the impact of healthy diet programs based on the Mediterranean diet pattern.

The Mediterranean diet has been found to reduce the risk of developing chronic diseases and the overall mortality rate in both experimental and observational studies [4,5]. The main explanation for the benefits of the Mediterranean diet is that it increases the intake of bioactive ingredients such as polyphenols, monounsaturated and polyunsaturated fatty acids, and fibers [33]. The increased adherence to the Mediterranean diet in the intervention group in our study, of about 3 points on a 14-point scale, was also higher than that observed in the PREDIMED trial, in which subjects in the intervention groups improved their scores by 2 points compared to no change in the low-fat control group [27]. This is possibly due to the inclusion of motivational counseling for the participants kept by the dietitians in our study, according to present educational theories [23], which has been shown to enhance adherence also in other studies [34].

The positive results of our trial, even in the control group, are noteworthy when considering the limits of lifestyle interventions among healthy adolescents. Indeed, participants in our trial were high school and university male students, most of whom lived with their parents and therefore had no active role, and probably limited influence, in meal preparation. Furthermore, since they were all healthy subjects, with normal blood pressure, blood parameters, and body mass index, they were less motivated to change their habits than obese or diseased people, who are usually the target of lifestyle interventions, although the volunteer participation in the trial has probably caused a self-selection of young people more interested in their own health than the general student population.

This RCT has some strengths, including the use of international standardized questionnaires, the multiple contacts between each participant, and an expert dietician in the intervention group for the whole period, the support of some organic food samples as an incentive to follow the dietician’s suggestions, and the relatively low attrition in the two arms, especially in the intervention group.

However, it has some limitations, too. The study design included only a short-term evaluation of the effects of the intervention; therefore, we cannot say if students’ lifestyle improvements will be maintained in the future. The observation at 4 months after the end of the intervention (t8) showed a trend of decreasing adherence to the Mediterranean diet and to a regular physical activity program. According to the main objective of the FASt project, i.e., evaluating the semen quality in adolescents living in polluted areas, only males were enrolled in our study. Therefore, a generalization of the study results to females may be seen as speculative. However, young females are usually more aware of health problems and at least as likely as peer males to follow dietary recommendations. Therefore, we are confident that our findings are of note for women too.

The randomization of subjects into the two arms of the study was correct, producing two comparable groups, as shown by the lack of differences between the intervention and control groups for almost all study variables at baseline. However, the RCT was not blinded, which is a limit of almost all dietary interventions, and therefore the assessment of participants’ lifestyle based on questionnaires may be prone to recall bias.

Third, a per-protocol analysis, including only the subjects with complete questionnaire data at each observation time, was performed. Even if an intention-to-treat analysis, including also subjects lost at follow-up, is usually performed in RCTs, such analysis was not feasible here, because the subjects withdrawn from the study did not attend the visit and questionnaire sessions. The dropout rates were higher in the control than the intervention group, at both visits, at the end of intervention time (17% and 7%, respectively), and at the end of the follow-up time (30% and 21%, respectively). However, the baseline characteristics of the individuals attending and those not attending the 4th- and 8th-month evaluations were not statistically different for almost all variables and dietary parameters. Therefore, we are confident that no substantial selection bias affected the results of the per-protocol instead of an intention-to-treat analysis, at least at the 4th-month evaluation.

## 5. Conclusions

In conclusion, this study has shown positive effects of a moderate short-term lifestyle intervention based on application of the Mediterranean diet principles and regular physical activity in healthy young men. These positive effects suggest that lifestyle, particularly dietary, interventions on adolescents and young people can be effective, provided that they are tailored to individual features and needs. At the same time, the decline in the adherence to dietary suggestions seen after the end of intervention underlines the need to evaluate the long-term effects of a lifestyle intervention.

## Figures and Tables

**Table 1:** Baseline characteristics of the participants.

Feature	INT (N = 66) Mean ± S.D.	CTRL (N = 63) Mean ± S.D.
age (years)	20.1 ± 1.2	20.0 ± 1.2
weight (kg)	70.7 ± 9.0	70.9 ± 9.3
height (cm)	177.5 ± 5.9	177.4 ± 5.8
BMI (kg/m^2^)	22.4 ± 2.2	22.5 ± 2.4
Waist circumference (cm)	83.1 ± 6.0	84.1 ± 5.8
Cigarette smokers N (%)	5 (7.6)	3 (4.8)
Occasional alcohol drinkersN (%)	44 (66.7)	48 (76.2)

One-way ANOVA for the comparison between the INT and CTRL groups: *p* > 0.20 for each.

**Table 2:** Food intakes measured by the EPIC-FFQ questionnaire at baseline (t0), end of intervention (t4), and end of follow-up (t8), in the intervention (INT) and control (CTRL) groups.

Variable	Group	t0 (Mean ± S.D.)	t4 (Mean ± S.D.)	t8 (Mean ± S.D.)	*p*^a^ (t4)	*p*^a^ (t8)
Vegetables (g/day)	INT	188.12 ± 118.23	208.39 ± 99.95	190.66 ± 76.22	<0.0001	0.0021
	CTRL	172.23 ± 118.24	165.66 ± 95.98	164.10 ± 114.61		
Onion and garlic (g/day)	INT	19.48 ± 13.33	17.07 ± 12.33	15.91 ± 10.56	0.8889	0.9719
	CTRL	18.19 ± 12.96	17.43 ± 14.27	16.14 ± 12.78		
Legumes (g/day)	INT	17.44 ± 14.32	36.1 ± 17.32	30.09 ± 17.77	<0.0001	0.0011
	CTRL	20.67 ± 20.48	24.62 ± 24.02	24.25 ± 20.87		
Fruits (g/day)	INT	273.11 ± 191.09	317.46 ± 150.02	295.88 ± 119.57	0.0003	0.0003
	CTRL	236.33 ± 161.35	245.95 ± 160.15	217.05 ± 121.76		
Nuts and seeds (g/day)	INT	9.52 ± 11.44	23.64 ± 9.11	18.70 ± 12.27	<0.0001	<0.0001
	CTRL	7.30 ± 8.68	8.71 ± 9.08	8.58 ± 9.25		
Whole grains (g/day)	INT	24.78 ± 32.37	50.92 ± 43.79	51.19 ± 47.71	<0.0001	<0.0001
	CTRL	30.23 ± 36.78	31.59 ± 44.96	28.00 ± 31.99		
Red meat (g/day)	INT	94.62 ± 47.21	58.09 ± 29.21	64.18 ± 35.94	<0.0001	0.0428
	CTRL	92.07 ± 58.86	82.70 ± 48.89	77.87 ± 44.43		
White meat (g/day)	INT	79.46 ± 55.06	69.77 ± 34.34	66.50 ± 38.92	0.3932	0.1421
	CTRL	78.81 ± 66.72	73.39 ± 57.09	64.54 ± 48.38		
Processed meat (g/day)	INT	52.81 ± 40.87	23.86 ± 21.61	27.39 ± 19.97	<0.0001	<0.0001
	CTRL	53.60 ± 34.98	51.71 ± 33.97	44.49 ± 32.01		
Fish (g/day)	INT	45.21 ± 26.65	73.80 ± 40.41	65.08 ± 31.05	<0.0001	0.0001
	CTRL	53.54 ± 38.18	50.10 ± 32.94	51.09 ± 36.53		
Olive oil (g/day)	INT	7.32 ± 5.92	8.44 ± 6.42	7.35 ± 6.05	0.7410	0.2707
	CTRL	8.04 ± 7.58	8.28 ± 7.29	9.12 ± 7.50		
EVO oil (g/day)	INT	16.77 ± 13.30	20.61 ± 13.02	18.65 ± 9.96	0.0553	0.0700
	CTRL	15.15 ± 9.19	15.95 ± 11.03	15.18 ± 11.33		
Total olive oil (g/day)	INT	24.09 ± 15.44	29.04 ± 14.73	26.00 ± 11.70	0.2465	0.5794
	CTRL	23.19 ± 11.32	24.23 ± 12.67	24.30 ± 11.80		
Butter (g/day)	INT	2.50 ± 4.12	1.94 ± 3.66	1.41 ± 2.23	0.5929	0.1821
	CTRL	3.05 ± 3.96	2.04 ± 2.85	2.03 ± 2.20		
Milk and yogurt (g/day)	INT	191.56 ± 149.39	179.39 ± 133.96	171.19 ± 126.60	0.3026	0.8195
	CTRL	171.77 ± 150.22	183.73 ± 150.43	185.90 ± 171.00		
Cheese (g/day)	INT	58.79 ± 53.70	40.33 ± 26.90	47.27 ± 33.54	0.0007	0.0662
	CTRL	72.81 ± 58.50	68.83 ± 40.41	69.28 ± 50.54		
Eggs (g/day)	INT	26.01 ± 16.17	25.36 ± 16.87	25.67 ± 14.67	0.4674	0.2772
	CTRL	27.24 ± 16.78	27.15± 17.35	24.62 ± 15.14		
Sweets (g/day)	INT	146.01 ± 129.71	91.87 ± 50.89	105.62 ± 78.53	0.0030	0.0020
	CTRL	91.41 ± 52.87	99.99 ± 68.74	100.92± 65.37		
Sweetened drinks (mL/day)	INT	207.43 ± 306.52	114.64 ± 104.54	112.43 ± 109.38	0.0144	0.0919
	CTRL	153.37 ± 157.80	132.91 ± 137.78	147.15 ± 124.11		
Coffee (mL/day)	INT	42.77 ± 45.64	42.10 ± 35.90	42.04 ± 35.22	0.8646	0.6877
	CTRL	40.93 ± 35.40	48.47 ± 39.61	50.17± 45.65		
Water (mL/day)	INT	1289.92 ± 534.29	1226.60 ± 327.03	1183.24 ± 313.38	0.9812	0.9507
	CTRL	1208.81 ± 324.55	1210.47 ± 332.20	1173.19 ± 334.82		

Number of participants in the groups: t0 and t4, INT: N = 66 and CTRL: N = 63; t8, INT: N = 60 and CTRL: N = 53. ^a^: ANCOVA of the differences between the two groups at t4 and t8.

**Table 3:** Macro- and micro-nutrient intake measured by the EPIC-FFQ at baseline (t0), end of intervention (t4), and end of follow-up (t8), in the intervention (INT) and control (CTRL) groups.

Variable	Group	t0 * (Mean ± S.D.)	t4 * (Mean ± S.D.)	t8 * (Mean ± S.D.)	*p*^a^ (t4)	*p*^a^ (t8)
Energy intake (Kcal/day)	INT	2923.56 ± 1070.16	2584.23 ± 663.96	2574.75 ± 797.21	0.0698	0.2481
	CTRL	2779.99 ± 807.53	2675.07 ± 628.95	2611.54 ± 702.96		
Total proteins (g/day)	INT	118.87 ± 39.01	102.76 ± 27.51	103.56 ± 32.22	0.0112	0.3274
	CTRL	121.11 ± 39.82	113.80 ± 31.35	109.88 ± 33.84		
Animal proteins (g/day)	INT	83.98 ± 32.74	66.90 ± 22.41	69.03 ± 24.84	0.0002	0.0796
	CTRL	86.30 ± 30.90	81.51 ± 26.69	77.93 ± 27.15		
Plant proteins (g/day)	INT	34.85 ± 10.78	35.79 ± 9.96	34.47 ± 11.05	0.0107	0.1967
	CTRL	34.74 ± 13.58	32.23 ± 9.82	31.90 ± 10.69		
Lipids (g/day)	INT	117.41 ± 46.02	107.72 ± 28.76	104.21 ± 32.67	0.2772	0.2348
	CTRL	112.90 ± 36.69	110.62 ± 30.47	107.70 ± 33.41		
Total saturated fats (g/day)	INT	39.41 ± 18.17	30.71 ± 9.81	31.55 ± 12.02	<0.0001	0.0050
	CTRL	38.69 ± 15.12	37.22 ± 11.99	36.18 ± 12.68		
Mufa (g/day)	INT	51.55 ± 19.42	50.44 ± 13.71	47.73 ± 14.35	0.8612	0.6122
	CTRL	49.32 ± 15.61	48.91 ± 13.92	47.64 ± 14.81		
Pufa (g/day)	INT	16.53 ± 6.15	16.93 ± 6.15	15.68 ± 5.01	0.1064	0.7006
	CTRL	15.59 ± 5.01	15.22 ± 4.48	14.78 ± 4.72		
Omega-6 fatty acid (g/day)	INT	13.04 ± 4.98	13.59 ± 5.41	12.53 ± 4.19	0.0371	0.5727
	CTRL	12.12 ± 4.08	11.91 ± 3.78	11.61 ± 4.01		
Omega-3 fatty acid (g/day)	INT	1.15 ± 0.38	1.29 ± 0.51	1.13 ± 0.36	0.0043	0.4409
	CTRL	1.12 ± 0.36	1.09 ± 0.33	1.07 ± 0.36		
Available glucides (g/day)	INT	363.73 ± 152.25	313.82 ± 93.98	317.89 ± 115.43	0.1771	0.3717
	CTRL	331.35 ± 112.86	314.61 ± 82.55	310.36 ± 89.00		
Soluble glucides (g/day)	INT	143.61 ± 89.32	121.51 ± 39.72	121.95 ± 51.82	0.2783	0.1980
	CTRL	112.81 ± 35.75	116.61 ± 44.11	113.57 ± 36.91		
Fibres (g/day)	INT	24.37 ± 9.22	28.20 ± 7.19	26.71 ± 8.34	<0.0001	0.0018
	CTRL	23.44 ± 7.20	23.17 ± 6.83	22.26 ± 6.27		
Sodium (mg/day)	INT	3047.87 ± 1382.52	2236.01 ± 754.85	2501.36 ± 977.73	<0.0001	0.0174
	CTRL	3232.32 ± 1210.10	3126.13 ± 1080.71	2903.83 ± 1072.99		
Vit C (μg/day)	INT	142.72 ± 66.59	158.02 ± 56.21	146.76 ± 47.77	0.0008	0.0398
	CTRL	131.98 ± 53.45	127.49 ± 48.95	126.09 ± 48.12		
Vit B6 (mg/day)	INT	2.42 ± 0.75	2.43 ± 0.84	2.30 ± 0.70	0.2765	0.3391
	CTRL	2.32 ± 0.68	2.23 ± 0.66	2.17 ± 0.71		
Folic acid (μg/day)	INT	345.26 ± 113.76	357.72 ± 103.72	344.01 ± 96.81	0.0134	0.0605
	CTRL	336.39 ± 94.66	318.06 ± 74.10	311.16 ± 80.75		
Retinol equivalents (μg/day)	INT	1244.94 ± 774.78	1268.41 ± 697.34	1251.35 ± 834.54	0.1019	0.1966
	CTRL	1165.99 ± 668.03	1098.38 ± 423.56	1081.51 ± 424.83		
Beta-carotene (μg/day)	INT	3899.63 ± 2504.85	4524.72 ± 2429.48	4039.25 ± 1851.24	0.0001	0.0192
	CTRL	3321.99 ± 1588.27	3298.04 ± 1644.56	3295.26 ± 1766.60		
Vit E (mg/day)	INT	16.12 ± 6.40	18.43 ± 5.74	16.84 ± 5.31	0.0002	0.0606
	CTRL	14.68 ± 4.35	14.80 ± 4.46	14.54 ± 4.49		
Vit D (μg/day)	INT	4.58 ± 2.04	4.59 ± 2.15	4.44 ± 1.77	0.7813	0.7114
	CTRL	4.53 ± 1.96	4.49 ± 1.90	4.35 ± 2.25		

* Number of participants in the groups: t0 and t4, INT: N = 66 and CTRL: N = 63; t8, INT: N = 60 and CTRL: N = 53. ^a^: ANCOVA of the differences between the two groups at t4 and t8.

## Data Availability

The data sets generated and/or analysed during the current study are not publicly available due to the sensitivity of data but are available from the corresponding author on reasonable request (elisabetta.ceretti1@unibs.it).
